# Investigating the Potential of Perovskite Nanocrystal-Doped Liquid Scintillator: A Feasibility Study

**DOI:** 10.3390/s23239490

**Published:** 2023-11-29

**Authors:** Na-Ri Kim, Kyung-Kwang Joo, Hyun-Gi Lee

**Affiliations:** Center for Precision Neutrino Research, Department of Physics, Chonnam National University, Yongbong-ro 77, Puk-gu, Gwangju 61186, Republic of Korea

**Keywords:** liquid scintillator, perovskite nanocrystal, wavelength shifter, PMT, neutrino detector

## Abstract

Liquid scintillators are extensively employed as targets in neutrino experiments and in medical radiography. Perovskite nanocrystals are recognized for their tunable emission spectra and high photoluminescence quantum yields. In this study, we investigated the feasibility of using perovskites as an alternative to fluor, a substance that shifts the wavelengths. The liquid scintillator candidates were synthesized by doping perovskite nanocrystals with emission wavelengths of 450, 480, and 510 nm into fluor PPO with varying nanocrystal concentrations in a toluene solvent. The several properties of the perovskite nanocrystal-doped liquid scintillator were measured and compared with those of a secondary wavelength shifter, bis-MSB. The emission spectra of the perovskite nanocrystal-doped liquid scintillator exhibited a distinct monochromatic wavelength, indicating energy transfer from PPO to the perovskite nanocrystals. Using a ${}^{60}\mathrm{Co}$ radioactive source setup with two photomultiplier tubes (PMTs), the light yields, pulse shape, and wavelength shifts of the scintillation events were measured. The light yields were evaluated based on the observed Compton edges from γ-rays, and compared across the synthesized samples. A decrease (or increase) in area-normalized PMT pulse height was observed at higher perovskite nanocrystal (or PPO) concentrations. The results demonstrated the sufficient potential of perovskite nanocrystals as an alternative to traditional wavelength shifters in a liquid scintillator.

## 1. Introduction

Liquid scintillators (LSs) are widely utilized as target materials in neutrino detectors [1,2] and medical applications [3]. Conventionally, LS are synthesized by mixing a solvent, such as toluene $\left(C_{6}H_{5}{\mathrm{CH}}_{3}\right)$[4,5] or pseudocumene $\left(C_{9}H_{12},\mathrm{PC}\right)$ [6] with 2,5-diphenyloxazole $\left(C_{15}H_{11}\mathrm{NO},\mathrm{PPO}\right)$ and 1,4-bis(2-methylstyryl) benzene $(C_{24}H_{22}$, bis-MSB) [7,8], which most commonly serve as a fluor and wavelength shifter, respectively. In recent decades, an alternative aromatic solvent, linear alkyl benzene ($C_{n}H_{2n+1}$-$C_{6}H_{5}$, n = 10–13, LAB) has been widely adopted due to its relatively high light yield, low toxicity, and environmentally friendly properties [9,10,11,12,13].

In the neutrino detector with a LS target, neutrino events are typically reconstructed based on the scintillation events observed by each photomultiplier tube (PMT) installed in the detector [14,15,16]. Numerous studies have been conducted to identify promising fluors for LS [1,17]. One candidate is perovskite nanocrystals for the wavelength shifter [18], which have been extensively studied for their potential applications in solar cells [19], light-emitting diode [20], and other photoelectronic devices [21]. The intriguing feature of perovskite nanocrystals is their narrow and tunable emission spectrum [22], making them suitable for these applications. However, to date, only a few attempts have been made to employ the quantum dots in neutrino or high-energy physics. The synthesis of perovskite nanocrystal-doped liquid scintillators (PVLS) and their associated light yield and emission spectra have been reported. Light yield behavior at low concentrations of perovskite nanocrystals has been measured [23], while another study documents light yields at higher concentrations [24]. Nonetheless, comprehensive measurements of scintillation event properties, such as light yield, pulse shape, and wavelength shift, have not yet been conducted as a function of nanocrystal concentrations in PVLS. In this work, we provide a direct comparison of several measured properties of PVLS with those of conventional wavelength shifters, such as bis-MSB-dissolved LS, to assess their potential.

We synthesized PVLS samples of various concentrations by dissolving commercially available perovskite nanocrystals [25,26,27] in a toluene solvent. The emission spectra of the PVLS samples were compared to those of the bis-MSB-dissolved LS samples at the same fluor concentration, which exhibited different emission wavelengths of 450, 480, and 510 nm. The features of the scintillation events, such as scintillation light yield, pulse shape, and emission spectrum shift, were measured using PMTs and a ${}^{60}\mathrm{Co}$ radioactive source for each LS samples. The scintillation light yield was estimated by fitting the Compton edges of γ-rays, and for each sample, the relative wavelength shift in the scintillation events induced by the quantum dots was evaluated using a PMT fitted with a 425 nm short-wavelength-pass filter. Evidently, the area-normalized pulse shape of the PVLS (or LS with only PPO dissolved in toluene) broadened (or sharpens) as the concentration of the perovskite nanocrystal (or PPO) increased, compared to that of the bis-MSB-dissolved LS. The observation indicates that PPO and perovskite nanocrystals can be employed as tools to adjust the pulse shape of a scintillation event [28]. We also report that superior corrected light yields can be achieved by replacing the wavelength shifter bis-MSB with a perovskite nanocrystal that emits photons at a wavelength of 510 nm.

## 2. The Synthesized PVLS and Emission Spectrum Measurement

The appearance and emission spectra of the synthesized PVLS were compared with those of PPO and the bis-MSB-dissolved LS. Toluene was first used in the LS as a solvent. Due to its high light yield and strong dissolving power, toluene were widely used as a basic solvent for fluor in the past [17]. Despite its advantages as an LS, research surrounding toluene-based LSs has been hesitant. Toluene, which possesses a benzene ring with a single carbon alkyl chain and is highly volatile, is harmful to the human body and environment [29]. However, in this study, we adopted toluene as a solvent owing to its strong dissolvability, which enabled the doping of perovskite nanocrystals [23]. Perovskite nanocrystals are a class of quantum dots with dimensions on the nanometer scale. When these materials are reduced to such sizes, their mobile charge carriers, such as electrons and holes, are effectively confined. This leads to a phenomenon called quantum confinement. As a result of this confinement, the crystal’s band gap increases as its size decreases. By adjusting the size of the perovskite nanocrystals, their emission wavelength can be controlled, allowing them to act as the wavelength shifters. Further details on using perovskite nanocrystals as wavelength shifters can be found in Ref. [23].

**Figure 1:**
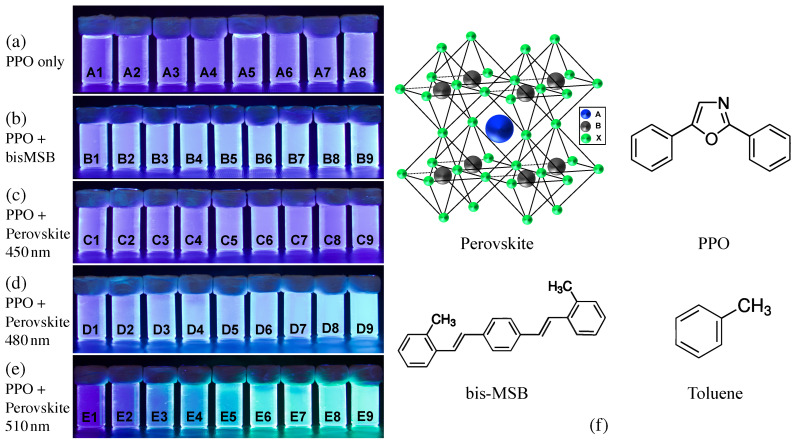
Scintillation light emitted by the PVLS samples under UV excitation: (**a**) eight samples with different PPO concentrations (PPO only); (**b**) nine samples with different concentration of bis-MSB (PPO fixed + bis-MSB); (**c**–**e**) nine samples with different concentrations of perovskite nanocrystals with 450, 480, and 510 nm (PPO fixed + perovskite). The PPO concentration is kept constant at 3 g/L for (**b**–**f**) Schematic structure of the perovskite solvent, fluor, and the conventional wavelength shifter used to synthesized the samples.

Initially, the LS composed of toluene and PPO was synthesized with different PPO concentrations as shown in Table 1. The PPO concentration ranged from 0.5 g/L to 10 g/L. The properties of the synthesized LS were measured and the PPO concentration of approximately ∼3 g/L was found to have the highest light yield. From this finding, the concentration of the primary fluor, PPO, was fixed at 3 g/L and samples with different types of wavelength shifters were synthesized. In the solution containing only perovskite nanocrystals, the presence of light yield was not apparent. Specifically, scintillation events could not be discerned without the dissolution of PPO. The commonly used bis-MSB and perovskite nanocrystal (perovskite quantum dots from Merck, Darmstadt, Germany) [25,26,27] with fluorescence emission wavelengths of 450, 480, and 510 nm were adopted as wavelength shifters in this study. Table 2 summarizes the bis-MSB and perovskite nanocrystal doped LS with different concentrations ranging from 0.5 to 50 mg/L at PPO concentration of 3 g/L. Figure 1a–e shows the photos for scintillation lights from the synthesized LS samples upon excitation with UV at 312 nm. The photo was captured by a digital camera (EOS D series from Canon, Tokyo, Japan). Figure 1f presents a schematic of toluene, PPO, bis-MSB, and perovskite nanocrystal structure for ${\mathrm{CsPbX}}_{3}\left(X=\mathrm{Cl},\mathrm{Br},I\right)$. The general formula of perovskite nanocrystal can be expressed as ${\mathrm{ABX}}_{3}$. In this structure, A represents a cation with an oxidation state of +1, B is a metal with an oxidation state of +2, and X denotes a halide with an −1 oxidation state [30].

**Figure 2:**
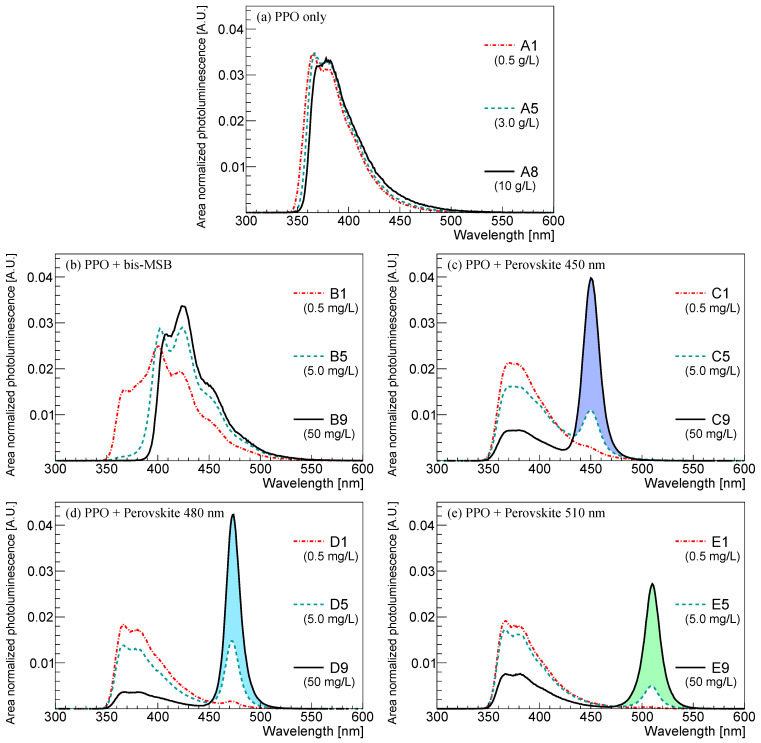
Emission spectra measurements of the synthesized LS samples shown in Table 1 and Table 2. Labels such as A1, A5, or A8 (or B1, B5, B9) refer to the samples with different concentrations of fluor (or wavelength shifter) listed in Table 1 (or Table 2): (**a**) Emission spectra for different PPO concentrations of 0.5, 3.0, and 10 g/L; (**b**) Emission spectrum with different bis-MSB concentrations; (**c**–**e**) emission spectra for different perovskite nanocrystal concentrations at 450, 480 and 510 nm. In (**b**–**e**), Spectra for wavelength shifter concentrations of 0.5, 5.0 and 50 mg/L. The PPO concentration was fixed at 3.0 g/L. The excitation wavelength for photoluminescence emission is 340 nm.

To analyze their optical properties in Table 1 and Table 2, the emission spectra of the synthesized LS samples with the different fluor concentrations were measured using a fluorescence spectrophotometer (Cary Eclipse fluorescence spectrometerfrom Varian Australasia, Sydney, Australia). Figure 2a presents the emission spectra of the PPO-only LS with different concentrations. The emission peak of PPO lies near a wavelength of 380 nm [9]. Compared to the spectra shown in Figure 1a–e, a slight shift in the emission spectra toward longer wavelengths is observed with the increasing fluor concentration of PPO. Similarly, the effects of the wavelength shifter are investigated at the fixed PPO concentration of 3 g/L. In Figure 2b, a distinct shift in the emission spectrum to higher wavelengths of around 420 nm is observed with the increasing bis-MSB concentration.

**Figure 3:**
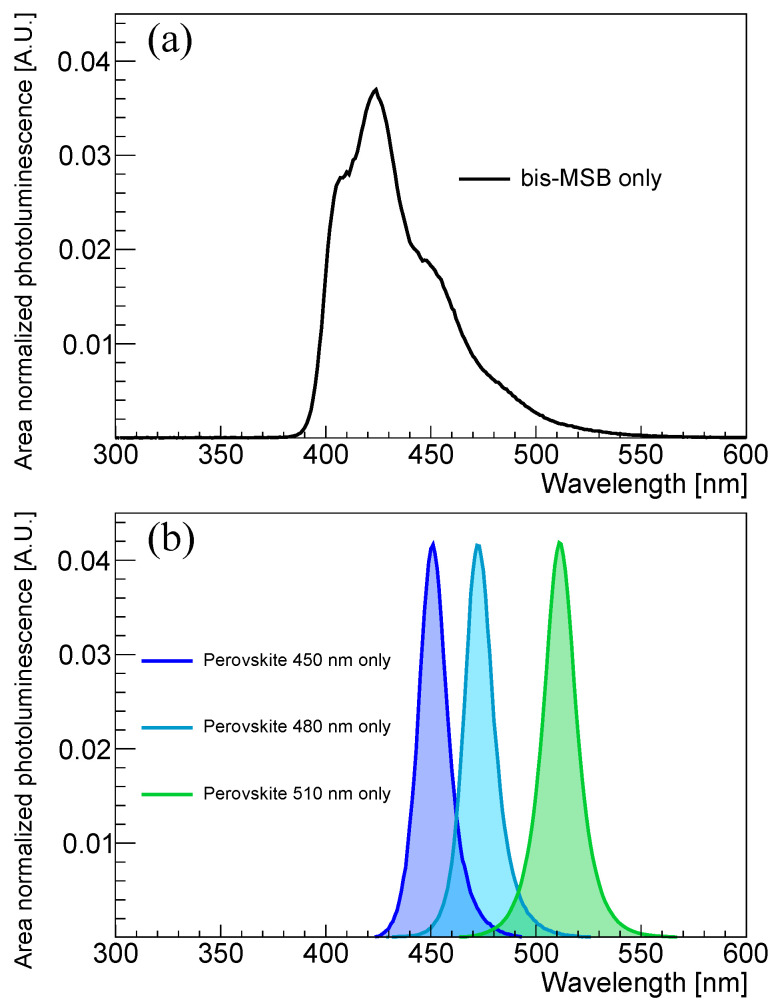
Emission spectra of bis-MSB and perovskite nanocrystals functioning as wavelength shifters. The wavelength shifter is dissolved in the solvent, toluene; (**a**) emission spectra of bis-MSB; (**b**) emission spectra of perovskite nanocrystals with 450, 480, and 510 nm. The color corresponding to each wavelength is shown as a translucent region.

Figure 2c–e show the emission spectra of the PVLS samples with varying concentrations of perovskite nanocrystals. As the concentration of the nanocrystals increases, the area-normalized photoluminescence intensity of the distribution at the monochromatic wavelengths around 450, 480, and 510 nm increases. Doping perovskite into PPO only LS does not change the monochromatic peak position due to the nanocrystal, while enhancing its relative emission intensity with their concentration. On the other hand, the emission intensity of PPO relatively decreases in the spectrum of the LS composite with PPO and perovskite, which suggests energy transfer from PPO to perovskite nanocrystals. Notably, the emission profile of bis-MSB shown in Figure 3a and that of the combination of PPO and bis-MSB are almost the same at high concentration. However, the emission spectrum of PPO combined with perovskite nanocrystals differs from that of the perovskite alone of Figure 3b at a wavelength shifter concentration of 50 mg/L. This difference may suggest that there is an unsaturated energy transfer from PPO to the perovskite at this concentration.

## 3. Scintillation Events Measurements Using Radioactive Source

**Figure 4:**
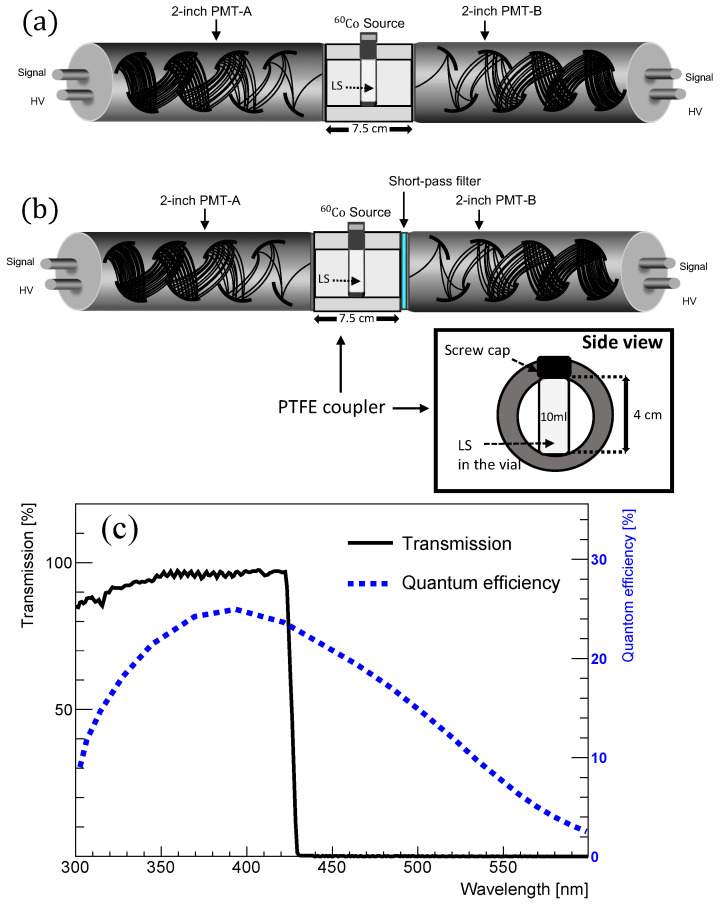
(**a**) Experimental setup used for measuring the scintillation light emitted from the synthesized samples. A vial containing the LS is situated in the center of the PTFE coupler. The response of the synthesized samples is measured using two adjacent 2-inch PMTs; (**b**) a short-pass filter is attached to the PMT on the right side to check for any shift in the emission spectrum; (**c**) quantum efficiency and transmittance values for the PMT H7195 and the 425 nm short-pass filter are depicted as a function of wavelength.

Variations in the emission spectrum with respect to fluor concentration were observed in the synthesized samples. To measure the scintillation event in the LS, a setup consisting of two PMTs and a radioactive source ${(}^{60}\mathrm{Co})$ was utilized, as illustrated in Figure 4a. The scintillation light, released from the samples through ionization, was detected by the PMTs as a current pulse. Additionally, a 425 nm short-pass filter was attached into the setup as depicted in Figure 4b to detect any relative shifts in the emission spectra of the scintillation events. In this configuration, various LS samples were measured to compare the light yield, pulse shape, and relative wavelength shifts of the scintillation events.

### 3.1. Experimental Setup

The measurement system containing two PMTs used to collect the scintillation light emitted from the LS samples is shown in Figure 4a. A 10 mL vial containing the synthesized samples was fixed in a cylindrical polytetrafluoroethylene (PTFE) coupler of 7.5 cm length with an inner diameter of ∼4 cm, which was located between the two facing PMTs and yielded the scintillation light from the Compton scattering of the γ-rays emitted by ${}^{60}\mathrm{Co}$. The gain of the two 2-inch diameter PMTs (H7195 from Hamamatsu, Shizuoka, Japan) [31,32] was adjusted to a typical gain of $∼3.0\times 10^{6}$.

The signals from the two PMTs were received by a flash-analog-to-digital-converter (FA DC 400 MHz from Notice, Seoul, Korea) with a 10-bit resolution to digitize the scintillation events. The PMTs are operated at 1600 V, provided by a high-voltage-module (ORTEC556 from ORTEC, Oak Ridge, TN, USA). The event triggering environment was set to be the coincidence of the two channels with a threshold of 4 mV. The coincidence condition of two PMTs is required for noise reduction. To identify the shift in the emission spectra, a 425 nm short-pass filter was attached to the 2-inch PMT-B as depicted in Figure 4b. The quantum efficiency (or transmittance) of the H7195 (or short-pass filter) is shown in Figure 4c with blue (or black) lines. The short-pass filter (high-performance optical density 4.0 short-pass filters from Edmund Optics, Barrington, IL, USA) had a diameter of $50.0_{-0.2}^{+0.0}$ mm.

### 3.2. Scintillation Light Yield Measurements

**Figure 5:**
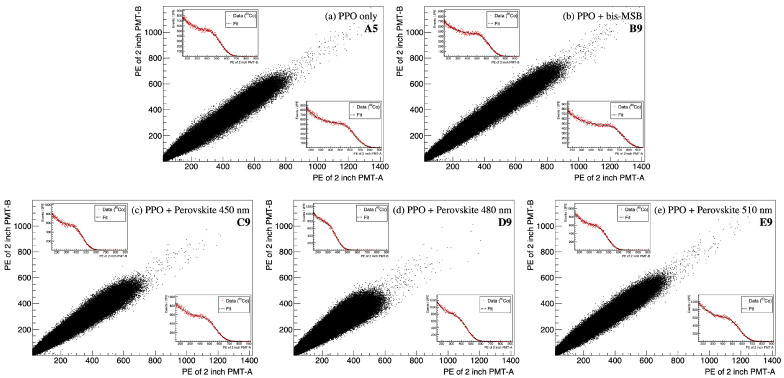
Observed scintillation light yield of the samples A5, B9, C9, D9, and E9, obtained from two H7195 PMTs using the setup shown in Figure 4a. A clear PE correlation is observed between the two PMTs by coincidence of the scintillation event. The inset presents a 1D projection plot for the observed PE. The upper (lower) inset corresponds to the PE distribution of PMT-B (PMT-A). Each PE distribution is fitted to characterize the light yield.

The scintillation light yield is an essential property for the energy resolution of the neutrino detector [1,33] and the spatial resolution of X-ray imaging for medical radiography [34]. The scintillation light yield of the synthesized samples is measured using a γ-ray source of ${}^{60}\mathrm{Co}$. The γ-rays emitted from the radioactive source transfer their energy through Compton scattering and the scattered electrons further ionize the LS samples. The scintillation light is detected with the two PMTs and is shown in Figure 5. The PMT-A and PMT-B observe the scintillation event through coincidence.

A clear correlation between the two PMTs for the observed number of photon electrons is found, irrespective of the types of LS samples. In Figure 5a,b, the toluene-based LS samples with a PPO concentration of 3 g/L (sample A9) and a concentration of 3 g/L of PPO and 50 mg/L of bis-MSB (sample B9) are shown. Figure 5c–e show a similar scatter plot for the PVLS, which is substituted for bis-MSB in the 450 (sample C9), 480 (sample D9), and 510 nm (sample E9) perovskite nanocrystal of 50 mg/L. To quantify the scintillation light yield, the distribution originating from the scattering of the γ-rays emitted by the ${}^{60}\mathrm{Co}$ source was fitted with an empirical function. The insets in Figure 5 show the observed photoelectron (PE) distribution of the scintillation events detected by the by PMT. The distribution is fitted to characterize the light yield. An empirical function of error plus exponential, $y=P_{0}\cdot \left[1-\mathrm{erf}\left(\left(x-P_{1}\right)/P_{2}\right)\right]+P_{3}\cdot \exp\left(-P_{4}\cdot x\right)$ is adopted to characterize the light yield of $P_{1}$ [35,36,37]. In this function, $P_{2}$ represents the resolution, $P_{0}$ denotes the distribution height, and $P_{3}$ and $P_{4}$ account for the added exponent for low PE. The scintillation light yield for each LS sample is defined by the summation of the obtained characterize the light yields of PMT-A and PMT-B.

### 3.3. Pulse Shape Measurements

**Figure 6:**
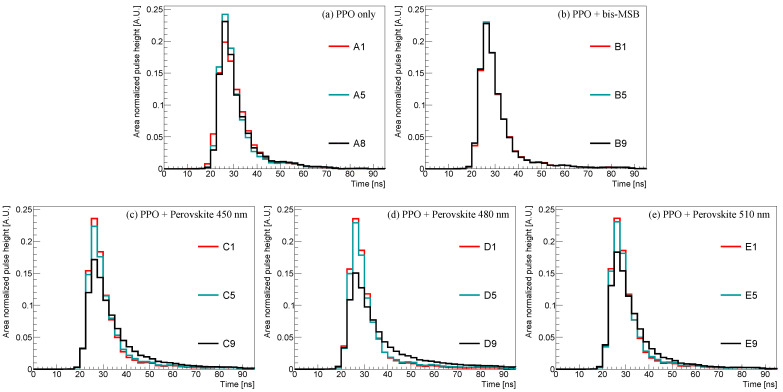
Area-normalized waveforms with different PPO, bis-MSB, and perovskite nanocrystal concentrations: (**a**) Observed pulse shape with varying PPO concentrations; (**b**) observed pulse shape with varying bis-MSB concentrations; (**c**–**e**) observed pulse shape with different perovskite quantum dot concentrations. Perovskite nanocrystals of 450, 480, and 510 nm doped in sample groups C, D, and E. Except for group A, the PPO concentration was constant (3 g/L). The waveform was obtained by averaging the area-normalized pulse shapes of 10,000 scintillation events.

The pulse of currents released from the PMT contains information about the particle types [38,39,40]. Figure 6 shows the accumulated pulse shape of scintillation events for varying fluor and the wavelength shifter concentration. As shown in Figure 6a, the difference in PPO concentration varies the observed pulse shape. The area-normalized pulse-height saturates at ∼3 g/L and slowly decreases for higher concentrations. However, in Figure 6b, the pulse shape variation is not evident for the bis-MSB concentration. In contrast, as shown in Figure 6c–e, the area-normalized pulse-height decreases as the concentration of the perovskite nanocrystals increases.

### 3.4. Relative Wavelength Shift Measurements

**Figure 7:**
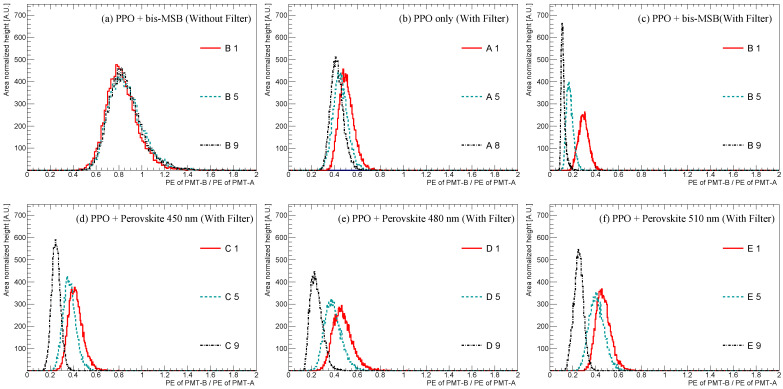
PE ratio observed between PMT-A and PMT-B using a 425 nm short-pass filter at different fluor concentrations: (**a**) measurement at the different bis-MSB concentration without filter for comparison; (**b**) measurement with different PPO concentrations; (**c**) measurement with different bis-MSB concentrations; (**d**–**f**) measurement with different perovskite nanocrystal concentrations of 450, 480, and 510 nm. The distribution shifts to the left as the concentration of the wavelength shifter increases. This shift is attributed to the use of the short-pass filter on the PMT-B side, which selectively allows wavelengths shorter than 425 nm to pass through, affecting the distribution. The PE ratio of (**b**–**f**) were obtained with the filter.

As presented in the previous section, the emission spectra of the synthesized samples vary with the concentrations of fluors and wavelength shifters: PPO, bis-MSB, and perovskite nanocrystals. The emission spectra variations in the scintillation events were demonstrated using the ${}^{60}\mathrm{Co}$-based experimental setup shown in Figure 4b; we compared the response of PMT-B, which was equipped with a 425 nm short-pass filter, with that of PMT-A without any filter. Figure 7 illustrates the ratio of the observed photoelectrons (PE ratio) between the two PMTs for the scintillation events emitted from the LS samples at different fluor concentrations. Discriminating the relative wavelength shift without the filter is challenging, irrespective of the PPO (3 g/L) and different bis-MSB concentrations as shown in Figure 7a. However, in Figure 7b–f, the distribution separates to the left side as the concentrations increase, indicating a relative shift in the scintillation spectra to wavelength larger than 425 nm.

### 3.5. Comparison of Observed Scintillation Properties

**Figure 8:**
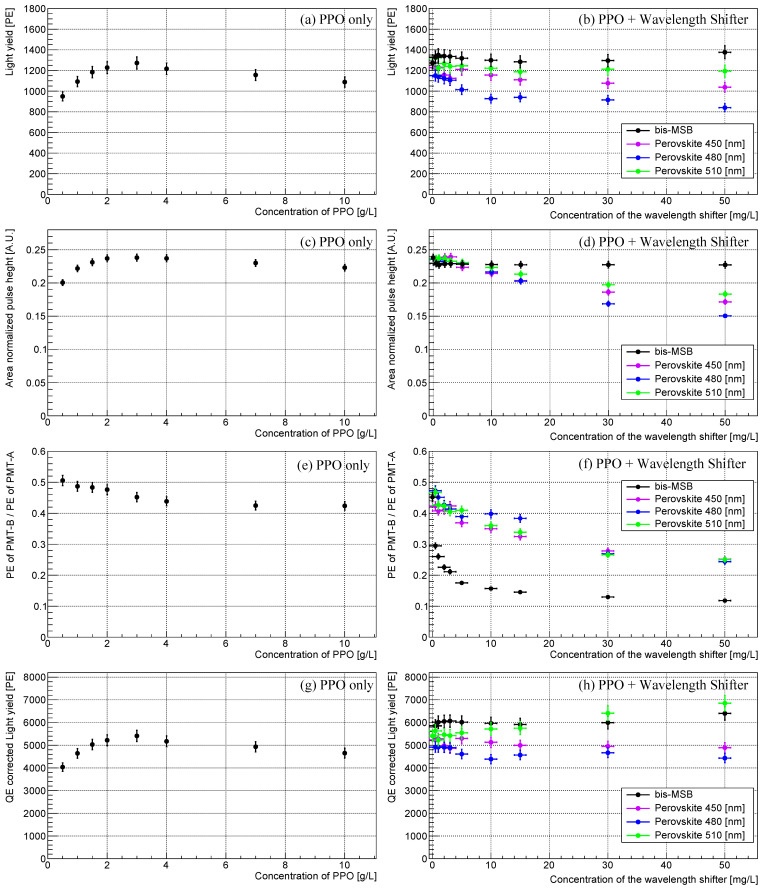
Featured properties of the synthesized LS samples with different PPO (wavelength shifter) concentrations are presented in (**a**–**e**). The scintillation light yield obtained from the Compton edge is presented in (**a**,**b**). (**c**,**d**) shows the area-normalized pulse-height obtained from the accumulated pulse height. The relative shift in the wavelength of the scintillation light is observed using a short-pass filter, as shown in (**e**,**f**). The QE-corrected light yield is given in (**g**,**h**). Uncertainties were determined from the deviations of values obtained from repeated measurements.

As described in the previous sections, we measured the scintillation events in the LS samples to analyze the properties of the LS, including scintillation light yield, pulse shape, and relative shift in the wavelength spectra at different concentrations of PPO and the wavelength shifters (bis-MSB and perovskite nanocrystals of 450, 480, and 510 nm). Figure 8 summarizes the obtained features for each synthesized sample as listed in Table 1 (varying PPO concentration) and Table 2 (varying wavelength shifter concentration).

Figure 8a,b show the obtained light yields of the synthesized LS samples with different fluor concentrations. The values are obtained through the sum of the Compton edges observed by PMT-A and PMT-B in the setup of Figure 4a. In Figure 8a, the light yield of the PPO-only LS saturates at around ∼3 g/L. Figure 8b shows the obtained light yields for the different types of wavelength shifters. For the bis-MSB case, the light yields slightly increase and saturate around ∼1 mg/L. The observed light yields obtained from the toluene-PPO-bisMSB-based LS are comparable to those used in RENO and Daya Bay [9,11]. The light yield of the PVLS decreases as the concentration increases. This trend may be attributed to differences in the quantum efficiency of the wavelength shifters [18,25,26,27], as well as PMT efficiency, as shown in Figure 4.

To account for the spectral sensitivity of PMT, a correction factor, denoted as $\varphi_{x}$ is introduced [24]. The $\varphi_{x}$ is defined as: $\varphi_{x}=\int \varphi \left(\lambda \right)I_{x}\left(\lambda \right)d\lambda /\int I_{x}\left(\lambda \right)d\lambda$ where, φ(λ) is the quantum efficiency of the PMT, H7195 for our study, and $I_{x}\left(\lambda \right)$ is the emission intensity at a given wavelength λ. The light yield corrected for PMT quantum efficiency (QE-corrected light yield), can then be obtained by dividing the original light yield by the correction factor $\varphi_{x}$. The Figure 8g,h depicts the corrected light yield, which eliminates the influence of wavelength-dependent PMT quantum efficiency. In contrast to Figure 8b, the wavelength independent light yields of PVLS with 510 nm emission gradually increase with nanocrystal concentration.

As mentioned before, the area-normalized pulse shapes for various PPO and perovskite nanocrystal concentrations (450, 480, and 510 nm) were analyzed. The peak of the area-normalized pulse shape, sensitive to the decay time of LS, is selected as a feature characteristic. This is illustrated in Figure 8c,d. Figure 8c exhibits the pulse-height for the PPO concentration. The heights saturate around 3 g/L and slowly decrease at higher concentrations. Figure 8d portrays the pulse heights for different types of wavelength shifters. In terms of bis-MSB concentrations, the decreases in pulse height saturate around 1 mg/L, but the effect is not conspicuous. Conversely, a clear decrease in height is observed for the PVLS samples, relative to the concentration. A difference in the decay time of the wavelength shifter—approximately 1.5 ns for bis-MSB and longer for perovskite nanocrystals—may account for this variation when added as a secondary fluor [18,41]. The decay time of perovskite nanocrystals, estimated from the pulse shape, is on the order of tens of nanoseconds.

The measured wavelength shift in the scintillation light is shown herein shift in the distribution of the PE ratio. The mean of the distribution, in accordance with the concentration, is shown in Figure 8e,f. A consistent decrease in the PE ratio has been observed for each sample, denoting a variation of the emission spectra of scintillation events to the higher wavelength due to the secondary emission from the wavelength shifter.

## 4. Discussion

In this study, we explored the possibility of using perovskite nanocrystals as alternatives to traditional wavelength shifters using the samples, whose details are shown in Table 1 and Table 2. We synthesized LS samples with varying PPO and perovskite nanocrystal concentrations (450, 480, and 510 nm) and compared their properties with those of LS samples dissolved in bis-MSB. The emission spectra measurements revealed a distinct monochromatic wavelength for the PVLS.

An increase in the concentration of perovskite nanocrystals resulted in a gradual shift in the average emission wavelength. Compared to bis-MSB, perovskite nanocrystals exhibit insensitivity to wavelength shifts. Perovskites could serve as tunable wavelength shifters; however, our observations imply that higher amounts of perovskite nanocrystals are required compared to bis-MSB. The scintillation events from the Compton scattering of the γ-rays were captured by the coincidence of PMT signal and a scintillation light yield, pulse shape, and emission spectrum shift are compared.

The light yield of the PVLS samples observed by the bi-alkali-photocathode PMT H7195 decreased with increasing perovskite nanocrystal concentration. However, in the case of perovskite nanocrystals emitting at 510 nm, the QE-corrected light yield gradually increased with concentration. This phenomenon was not observed with perovskite nanocrystals emitting at 450 and 480 nm. This discrepancy can be explained by their relatively low quantum yields at these shorter wavelengths, as indicated in the specification sheet [25,26,27].

The area-normalized pulse-height released from the PMT decreased as the concentration of perovskite increased, which may be attributed to the relatively long decay time of the perovskite nanocrystals compared to bis-MSB. This high concentration of perovskite nanocrystals extends the PMT pulse shape. The wavelength shift in the scintillation events was also observed using a PMT combined with a short-pass filter.

The results obtained in this study demonstrate the feasibility of using perovskite nanocrystals as alternatives to traditional wavelength shifters in LS. Future studies could be focused on enhancing the quantum efficiency of perovskite nanocrystals [42] or to optimize quantum efficiency in the perovskite emission region using green-sensitive photosensors [43] to enhance the performance of neutrino detectors and other devices. Additionally, further studies on the long-term stability [44], the radiation hardness [45], and temperature performance [46] of PVLS are necessary for their successful implementation in practical applications.

## Figures and Tables

**Table 1:** Synthesized LS samples with different PPO concentrations from 0.5 g/L to 10 g/L. The perovskite nanocrystals or wavelength shifter were not contained in the samples.

Sample	PPO (g/L)
A1	0.5
A2	1
A3	1.5
A4	2
A5	3
A6	4
A7	7
A8	10

**Table 2:** Synthesized LS samples with different wavelength shifter concentrations. The concentration of PPO was fixed at 3 g/L, while that of the concentration of the wavelength shifter was varied from 0.5 mg/L to 50 mg/L. The wavelength shifter bis-MSB was dissolved in the samples B1–B9, and the 450, 480, and 510 nm perovskite nanocrystals were doped in the samples C1–C9, D1–D9, and E1–E9, respectively.

Sample				PPO (g/L)	Wavelength Shifter (mg/L)
bis-MSB	Perovskite 450 nm	Perovskite 480 nm	Perovskite 510 nm		
B1	C1	D1	E1	3	0.5
B2	C2	D2	E2	3	1
B3	C3	D3	E3	3	2
B4	C4	D4	E4	3	3
B5	C5	D5	E5	3	5
B6	C6	D6	E6	3	10
B7	C7	D7	E7	3	15
B8	C8	D8	E8	3	30
B9	C9	D9	E9	3	50

## Data Availability

The datasets generated or analyzed in this study are available from the corresponding authors on reasonable request.
